# Ultra-processed foods consumption among a USA representative sample of middle-older adults: a cross-sectional analysis

**DOI:** 10.1017/S0007114523003033

**Published:** 2024-04-28

**Authors:** Abeer Ali Aljahdali, Sinara Laurini Rossato, Ana Baylin

**Affiliations:** 1 Department of Clinical Nutrition, Faculty of Applied Medical Sciences, King Abdulaziz University, Jeddah, Saudi Arabia; 2 Department of Nutritional Sciences, School of Public Health, University of Michigan, Ann Arbor, MI, USA; 3 Department of Nutrition, Harvard T.H. Chan School of Public Health, Boston, MA, USA; 4 Institute of Geography, Universidade Federal de Uberlândia, Uberlândia, MG, Brazil; 5 Department of Global Public Health, University of Michigan, Ann Arbor, MI, USA

**Keywords:** Food processing, Ultra-processed foods, Nova, Diet quality, Nutrient profile, Health and Retirement Study (HRS), Middle-older adults, USA

## Abstract

The study evaluated the association between ultra-processed foods (UPF) and nutrient intake and identified the socio-demographic characteristics associated with UPF consumption among a nationally representative sample of middle-older adults. Dietary assessment was collected in 2013 using a validated FFQ. The Nova system was used to classify food and drinks into UPF. The percentage of dietary energy from UPF was calculated and used throughout the analyses, and average nutrient intake across quintiles of UPF was evaluated. The determinants associated with the dietary caloric contribution of UPF intake were investigated using linear regression models. A cross-sectional analysis of a nationally representative study of Americans over the age of 50, the Health and Retirement Study, was conducted. The analysis included 6220 participants. The mean age was 65 (se 0·28) years, with 55 % being female. UPF intake accounted for 51 % (se 0·25) of total intake. An increase in the percentage of (%UPF) consumption was correlated with an increase in calories, carbohydrates, saturated fat and sugar, and a decrease in fibre, vitamins and minerals. %UPF intake was inversely associated with being Hispanic, higher income, physical activity, vegetarian diet and Mediterranean diet but positively associated with very low food insecurity. UPF represented half of the calories consumed. A higher %UPF intake was associated with a lower nutrient profile, suggesting decreasing %UPF intake as a strategy to improve the nutritional quality of middle-older adults. A few socio-demographic factors were associated with %UPF, which would help in planning strategies to reduce UPF consumption.

An ageing population is a global phenomenon, and older adults share a significant proportion of the global population. The proportion of older adults and their life expectancy have increased in the USA during the past decade^(1,2)^. In 2016, adults aged ≥ 65 years represented 15 % of the USA population, and it is expected to reach 21 % by 2030^(1,2)^. Ageing is associated with physiological changes that may result in impaired functional abilities and increased morbidity and mortality^(3)^. The concept of successful ageing has emerged to describe the quality of ageing^(4)^, and it aims to lower morbidity while optimising social engagement and functional abilities^(5)^. Because of its critical role in healthy ageing^(6)^, healthy nutrition is a lifestyle-preventive approach to promoting healthy ageing^(6)^.

Multiple diet-related concerns have been reported among USA older adults. For example, Choi et al. showed that only 11 % of USA adults aged ≥ 54 years had optimal diet quality assessed using the Healthy Eating Index^(7)^. Similarly, there is a documented decline in diet quality between 2001 and 2018 by nearly 8 % among USA older adults aged ≥ 65 years enrolled in the National Health and Nutrition Examination Survey^(8)^. Also, food insecurity, defined as ‘the limited or uncertain availability of nutritionally adequate and safe foods, or the limited or uncertain ability to acquire acceptable foods in socially acceptable ways’^(9)^, has been reported as another nutritional concern for USA older adults^(10,11)^. Positive trends for food insecurity among USA older adults aged ≥ 65 years have been reported since 1999^(11)^. Previous research found a link between diet quality and food insecurity; it was shown that food-insecure older adults had lower diet quality than food secure counterparts^(7,11)^.

Multiple approaches have been used to assess diet quality in population research. The Nova classification system is a major food processing classification in the scientific literature^(12)^, and it has been endorsed by the FAO of the UN^(13)^. According to the extent, nature and purpose of food processing (i.e. physical, biological and chemical processes), food is classified into four distinct groups: (1) unprocessed and minimally processed foods, (2) processed culinary ingredients, (3) processed foods and (4) ultra-processed foods (UPF)^(13–15)^. Food processing may have both positive and negative consequences^(13,16)^; one of its well-documented purposes is to make convenient and palatable foods at a lower cost than home-prepared foods from fresh ingredients^(13–15)^. The Nova classification was not only introduced in light of the global food system transformation^(13)^ but also because food processing, and its potential health implications were neglected in most of the previous USA population dietary recommendations^(13,14)^.

Characterising the diet according to the degree of food processing among older adults is of great interest given the growing body of evidence supporting the relationship between UPF and adverse health outcomes among older adults^(17–24)^. Previous studies have shown that UPF consumption has been positively associated with frailty^(17–20)^, impaired cognitive function^(21,22)^, higher risk of dementia^(23)^ and sarcopenia^(24)^. However, there are no prior studies on the association between UPF and diet quality among USA middle-older adults. Thus, this study aimed to evaluate the association between UPF and nutrient intake among a nationally representative sample of middle-older adults in the USA. Also, the socio-demographic and lifestyle characteristics associated with UPF consumption were identified to provide knowledge for public health policy and interventions for promoting healthy nutrition. Because UPF consumption has been previously reported to be higher among men^(25–28)^, sex heterogeneity in UPF consumption was explored.

## Methods

### Sample and data

The Health and Retirement Study (HRS) is a nationally representative longitudinal survey of USA adults aged 50 and older. The HRS cohort profile is described in detail elsewhere^(29)^ and is available at the HRS website http://hrsonline.isr.umich.edu. In brief, a core survey of an interviewer-administered interview was completed every 2 years on income and wealth, health and use of healthcare services, work and retirement and family connections, and a series of physical measurements were obtained^(29)^. In addition, participants are also invited to enroll in supplementary studies between the core waves^(29)^. One of these was the 2013 Health Care and Nutrition Study (HCNS), which inquired about health care access, food purchases and consumption and nutrition, including vitamins and supplements, using mailed questionnaires sent to a random sample of HRS respondents and their spouses/partners (*n* 12 418)^(30,31)^. A validated FFQ was used to quantify consumption over the past year for the survey’s respondents^(32)^. The HCNS was conducted from November 2013 to May 2014. Out of the invitations sent, only 8073 respondents completed the survey, which resulted in a 65 % simple response rate^(30,31)^. Among these, 97 % had completed 90 % of the FFQ questions, and missing information was imputed^(31)^. The other 3 % of the respondents had completed less than 3 % (*n* 37) of the FFQ, and there was a respondent who was on tube feeding (*n* 1); these respondents were excluded (*n* 37)^(31)^.

**Fig. 1.**
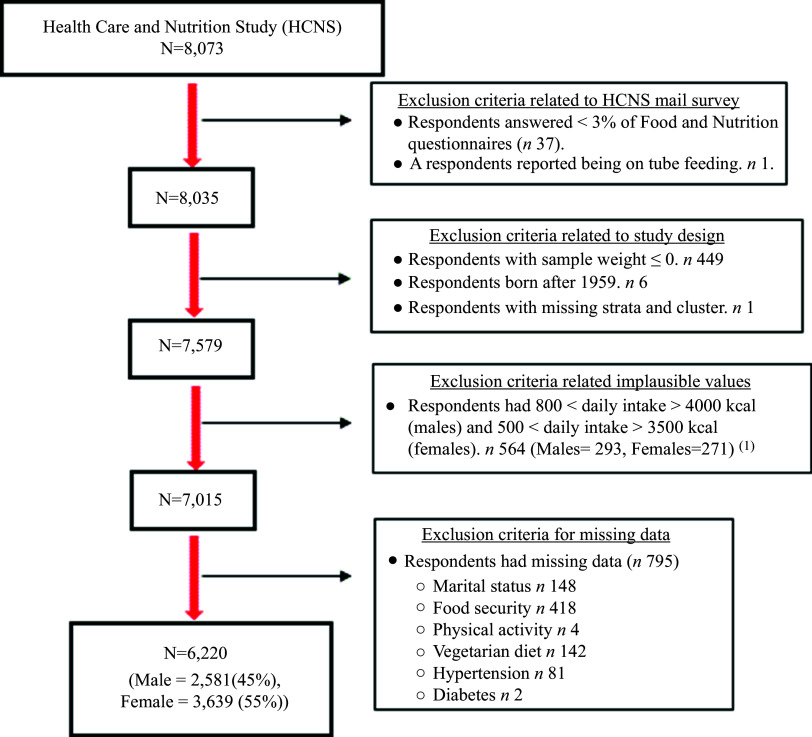
Flow chart of the exclusion criteria. Footnote 1. Pimenta AM, Toledo E, Rodriguez-Diez MC, Gea A, Lopez-Iracheta R, Shivappa N, et al. Dietary indexes, food patterns and incidence of metabolic syndrome in a Mediterranean cohort: The SUN project. Clin Nutr. 2015;34(3):508–14.

This cross-sectional analysis includes participants who completed a core HRS interview in 2012 and the supplemental HCNS in 2013. A complete case analysis approach was followed; thus, of these participants enrolled in the HCNS (8073), only *n* 6220 (male = 2581 and female = 3639) had non-missing data for variables of interest were included in this study. Figure 1 illustrates the exclusion criteria and the final sample size.

HRS is funded by the National Institute of Aging (NIA U01AG009740) and the Social Security Administration and is conducted by the Institute of Social Research at the University of Michigan. This study was conducted according to the guidelines laid down in the Declaration of Helsinki, and all procedures involving research study participants were approved by the University of Michigan Health Sciences/Behavioral Sciences Institutional Review Board (IRB) (Protocol: HUM00061128). Written informed consent was obtained from all subjects/patients.

### Measures

#### Assessment of the foods, beverages and nutrient intake

During the HCNS, a validated FFQ^(33)^ was mailed to all eligible subjects in Fall 2013 to assess the previous year’s food and beverage consumption pattern. The FFQ contained 163 food and beverage items and had eight frequency options, which were ‘never, < 1 time/a month, 1–3 times/ a month, once/ a week, 2–4 times/ a week, 5–6 times/ week, once/ a day and ≥ 2 times/ a day.’ Each food and beverage item included one fixed portion size option. The HRS team cleaned the collected HCNS data^(30)^ and computed the average daily consumption for each FFQ item based on the selected categorical response out of the eight frequency options^(31)^. The calories and nutrient analysis for each item were calculated by the study team by matching the daily servings of FFQ items with relevant food and beverage items listed in the Harvard School of Public Health nutrient composition tables^(34)^.

#### Classification of foods and beverages into the Nova groups

The Nova system categorises foods and beverages into four distinct foods groups based on the nature, extent and purposes of the industrial processes^(14,15)^. Unprocessed and minimally processed foods are the edible parts of plants or animals, and natural foods altered by any of the following methods: removal of inedible or unwanted parts, drying, crushing, grinding, powdering, fractioning, filtering, roasting, boiling, non-alcoholic fermentation, pasteurisation, chilling, freezing, placing in containers and vacuum packaging. Processed culinary ingredients include substances derived from unprocessed and minimally processed foods or nature via pressing, refining, grinding, milling and drying. Processed foods are prepared by adding salt, oil, sugar or other processed culinary ingredients to unprocessed and minimally processed foods and include bread, cheeses and other non-alcoholic fermented foods^(14,15)^. UPF are ready-to-consume foods manufactured through techniques designed for industrial settings and generally contain artificial substances to preserve and enhance flavour, colour or presentation^(14,15,35)^. Each of the FFQ items was categorised into a mutually exclusive Nova group (i.e., UPs, processed culinary ingredients, processed foods or UPF) following the approach published by Rodrigues et al., herein called Classification 1^(36)^. As sensitivity analysis in classifying FFQ items to Nova group, the classifications proposed by Khandpur and colleagues (sensitivity 1, 2 and 3)^(37)^ were followed. The research team also proposed an additional classification (sensitivity 4) (online Supplementary Table 1). The same conclusions were derived from the sensitivity analyses, so only results from classification 1 were presented. The 164 foods and beverages listed in the FFQ were further grouped in each Nova classification into sub-groups based on their nutritional properties (online Supplementary Table 2). The study focused on the UPF group, and the dietary caloric contribution of UPF was used for the analysis in the current study.

#### Covariates

Self-reported socio-demographic characteristics and health and lifestyle-related information were collected during the core HRS and HCNS. Age at the HCNS (2013) was used in this analysis. The race variable was categorised into White, Black, Hispanic or others – including American Indian Alaskan Native, Asian, Native Hawaiian, Pacific Islander and others. Marital status was assessed as a categorical variable: married, separated/divorced/widowed and never married. Years of education completed were classified into four categories as follows: (less than high school graduate (0–11 years), graduated from high school (12 years), some college or college graduate (13–16 years) or post-college (≥ 17 years)). Total net wealth, which includes all assets except secondary residence^(38)^, was used to assess the financial status. Smoking status was categorised into current smoker or nonsmoker. Physical activity was classified as hardly ever or never doing moderate or vigorous activities, a few times per month and once a week or more). BMI was categorised into four groups: underweight (< 18·5 kg/m^2^), normal weight (18·5–24·9 kg/m^2^), overweight (25·0 – 29·9 kg/m^2^) and obese (≥ 30·0 kg/m^2^). Following a vegetarian diet was coded as a binary variable. Receiving a diagnosis by a medical professional or using medication(s) to control diabetes (yes/no), hypertension (yes/no) or any heart diseases (heart attack, coronary heart disease, angina, congestive heart failure, congestive heart failure, abnormal heart rhythm or stroke) was assessed (yes/no). Receiving free or subsidised foods in the last year from foods bank/foods pantry, church, shelter, meals on wheels, senior brown bag or other home-delivered meal services or other sources of foods donations was coded as a binary variable. Food security status was assessed using the Six-Item Short Form of the Food Security Survey Module. The summed responses’ raw scores ranged from 0–6 and were categorised into: food security – raw score 0–1; low food security – raw score 2–4 and very low food security – raw score 5–6^(39)^. The alternate Mediterranean Diet (aMedDiet) score was calculated^(40,41)^ as a dietary index for assessing the overall diet quality. In summary, the aMedDiet score is the sum of nine food groups binary variables. If the intake of fruits, vegetables, whole grains, nuts, legumes, fish and the ratio of MUFA to SFA exceeded the sex-specific median intake values of the study population, or if the intake fell short of the study population’s sex-specific median intake values of red/processed meats, a score of one was assigned. For males and women, respectively, moderate alcohol intake was rated as 0·5–2 and 0·5–1·5 drinks per day, with a score of one. As a result, aMedDiet score has a range of zero to nine, with higher numbers denoting greater adherence to a Mediterranean-style diet^(40,41)^.

### Statistical analysis

The consumption of UPF was quantified and expressed as a %UPF. The associations between the dietary caloric contribution of UPF and socio-demographic characteristics and nutrient intake were explored using a sex-stratified analysis. Data were expressed as means (standard error (se)) or means (95 % CI). Only participants who had no missing data on the variables of interest were included in this analysis. All statistical analyses accounted for HRS’s complex, multistage, stratified, cluster-sampling design using sample weights, strata and primary sampling units released with the HRS data.

The association between %UPF and nutrient intake was conducted by comparing the overall average nutrient content across quantiles of %UPF. Nutrients of interest were expressed as kilocalories (kcal) or μg/mg/gm/μg per 1000 kcal. The following macronutrients and micronutrients were assessed: calories, carbohydrate, protein, fat, SFA, fibres, added sugars, vitamin A, folic acid, vitamin B_12_, vitamin C, vitamin K, Ca, Fe, Mg, phosphorus, potassium and Na. The inclusion of covariates in adjusted models was based on the significance of the bivariate association between %UPF and the covariates, presented in Table 1. Bivariate associations were examined using independent *t* tests to detect between-group differences in the mean dietary intake of %UPF for binary covariates or linear regression models for categorical covariates. Age was adjusted for in these models to account for the age heterogeneity in dietary intake regardless of the significance of its crude association. We assessed the linear association between 10 % increase in the %UPF and each nutrient. Also, we examined the linear trend by modelling quantiles of %UPF as an ordinal variable in linear regression models. Quantiles-based categorisation of the exposure variable was used to evaluate dose–response relationships and detect any non-linear associations.

**Table 1.** Mean dietary caloric contribution of ultra-processed foods group by socio-demographic and psychosocial characteristics among health and retirement study population

	Males			Females		
	*n*		2581	*n*		3639
	%		45·34	%		54·66
	UPF†		*P* value	UPF†		*P* value
	Mean	95 % CI		Mean	95 % CI	
Age (years)‡			0·2115			0·3292
< 65	53	52·5, 54·3		50	48·9, 50·9	
≥ 65	53	51·9, 53·3		50	49·8, 51·2	
Race, %§			< 0·0001***			< 0·0001***
White (Ref.)	54	52·9, 54·2	Ref.	51	50·3, 51·7	Ref.
African American	53	51·3, 55·1	0·7388	49	48·0, 50·9	0·0633
Hispanic	48	46·5, 50·5	< 0·0001***	45	43·0, 46·4	< 0·0001***
Other	49	44·3, 53·4	0·0596	45	42·8, 47·6	0·0003***
Marital status, %§			0·0557			0·2125
Married (Ref.)	53	52·0, 53·2	Ref.	50	49·0, 50·6	Ref.
Separated/divorced/widowed	54	52·1, 55·3	0·2067	51	49·8, 51·7	0·1011
Never married	55	53·2, 57·7	0·0198*	50	47·7, 52·2	0·9273
Education level, %			0·0007***			< 0·0001***
Less than high school (Ref.)§	53	51·9, 54·6	Ref.	51	48·9, 52·3	Ref.
High school grad	55	53·4, 55·7	0·1237	52	51·3, 53·0	0·0967
Some college/ grad	53	51·8, 53·7	0·5528	50	48·4, 50·7	0·3615
Post-college	51	49·6, 52·1	0·0123*	47	45·2, 48·3	0·0028**
Total net wealth (USA $)§			< 0·0001***			< 0·0001***
–1 510 000–25 150 (Ref.)	56	54·1, 57·2	Ref.	51	50·2, 52·7	Ref.
25 500–146 750	54	52·9, 54·9	0·0532	51	50·1, 52·7	0·9697
146 889–442 424	53	52·1, 53·8	0·0034**	51	49·8, 51·7	0·4403
442 500–22 114 000	51	50·0, 51·8	< 0·0001***	47	46·4, 48·6	< 0·0001***
Food security, %§			0·0060**			0·0081**
Food security (Ref.)	53	52·0, 53·3	Ref.	50	49·0, 50·6	Ref.
Low food security	54	51·4, 55·9	0·4131	51	48·5, 52·6	0·5391
Very low food security	57	54·7, 59·7	0·0016**	54	51·5, 56·3	0·0021**
Receiving free or subsidised food, %‡			0·2141			0·0333*
No	53	52·3, 53·5		50	49·2, 50·7	
Yes	54	52·4, 55·6		52	50·3, 53·3	
Physical activity, %§			< 0·0001***			< 0·0001***
Hardly ever or never (Ref.)	57	54·8, 58·5	Ref.	54	52·2, 54·9	Ref.
A few times per month	56	54·0, 57·4	0·5074	53	51·8, 54·6	0·7021
Once a week or more	52	51·4, 52·9	< 0·0001***	49	48·1, 49·5	< 0·0001***
Smoking status, %‡			0·0013**			< 0·0001***
No	53	52·1, 53·1		50	48·9, 50·2	
Yes	56	53·8, 57·5		55	53·5, 56·6	
BMI, %§			0·2306			0·0006**
Underweight	43	28·6, 57·4	0·1701	53	50·1, 56·5	0·1060
Normal weight (Ref.)	53	51·4, 54·5	Ref.	49	48·1, 50·6	Ref.
Overweight	53	51·5, 53·5	0·6054	49	48·1, 49·9	0·5756
Obese	54	52·8, 54·4	0·4272	51	50·5, 52·3	0·0062**
Vegetarian diet, %‡			0·0008***			< 0·0001***
No	53	52·6, 53·8		51	49·9, 51·2	
Yes	45	41·0, 48·8		42	38·8, 45·9	
Alternate Mediterranean diet, %‡,||			< 0·0001***			< 0·0001***
Below or equal the median cut-off value	57	56·1, 58·1		55	54·4, 56·3	
Above the median cut-off value	51	50·1, 51·4		47	46·4, 47·8	
CVD, %‡			0·0002***			0·0465*
No	52	51·8, 53·1		50	49·1, 50·6	
Yes	55	53·6, 55·7		51	50·2, 52·4	
Hypertension, %‡			0·7699			0·0298*
No	53	52·0, 53·9		50	48·6, 50·4	
Yes	53	52·4, 53·8		51	50·0, 51·4	
Diabetes, %‡			0·0454*			0·0908
No	53	52·6, 54·0		50	49·7, 51·0	
Yes	52	50·8, 53·1		49	48·0, 50·5	

UPF, ultra-processed foods; TEI, total daily energy intake; Ref., reference.

* *P* < 0·05.

** *P* < 0·01.

*** *P* < 0·001.

† Means (95 % CI) are presented.

‡ Independent sample *t* test was used.

§ Linear regression was used.

|| Sex-specific medians cut-off were used to dichotomise the alternate Mediterranean diet score; for men the cut-off was 3·78 and for women the cut-off was 3·75.

Furthermore, determinants of %UPF consumption were explored using linear regression. Crude and fully adjusted modes were presented, and the beta estimate (*β*) with respective 95 % CI were provided. In the fully adjusted models, we included all socio-demographic and psychosocial characteristics to determine the most predicting covariates. Statistical tests were two-sided at a significance level of 0·05. Statistical Analysis Systems (SAS) software (SAS Institute Inc.), version 9.4, was used for all analyses.

### Results

The analysis included 6220 participants of the HCNS, 81 % were White Americans, with an average age of 65 (se 0·28) years (52–101 years). Table 1 shows the dietary caloric contribution of UPF across demographic characteristics and health behaviour variables among the study population. There was a crude significant difference in mean %UPF across races, where Hispanics showed lower intake than White Americans (i.e. reference group) in both sexes. However, among females only, participants who belonged to another race group had a lower intake 45 (95 % CI 42·8, 47·6) compared with the reference group 51 (95 % CI 50·3, 51·7) (*P* value = 0·0003). Education, physical activity, income and adherence to vegetarian or Mediterranean diet patterns were inversely associated with %UPF in both sexes (all *P* values < 0·001). Very low food security was associated with higher %UPF in both sexes compared with the food-secured reference group (all *P* values < 0·001). Receiving free or subsidised foods and higher BMI were associated with higher %UPF among females (all *P* values < 0·05). Smoking and CVD were crudely associated with higher mean %UPF in both sexes (all *P* values < 0·05). However, hypertension was positively associated with %UPF intake, 51 (95 % CI 50·0, 51·4) compared with the reference group 50 (95 % CI 48·6, 50·4) (*P* value = 0·0298) in females, but diabetes was inversely related with %UPF, 52 (95 % CI 50·8, 53·1) compared with the reference group 53 (95 % CI 52·6, 54·0) (*P* value = 0·054) among males (Table 1).

Sex-stratified means dietary share of the Nova groups are presented in Table 2. A higher consumption of %UPF was reported among males (53 % (95 % CI 52·4, 53·6) compared with females (50 % (95 % CI 49·6, 50·8). After categorising the FFQ items into seventeen subgroups based on their nutritional characteristics, the four highest groups contributing to the consumption of %UPF were identified. These groups were (1) bread and breakfast foods (10 %), followed by (2) candies, chocolate and flour-based sweets (10 % in males and 9 % in females), (3) processed animal protein (8 % in males and 7 % in females) and (4) snack and other savory foods (7 %). The subgroups comprising the UP, processed culinary ingredients and processed foods shares are presented in online Supplementary Table 3.

**Table 2.** Mean dietary caloric contribution Nova groups and subgroups among health and retirement study population

	Males				Females			
	*n*		2581		*n*		3639	
	%		45·34		%		54·66	
	% of TEI		% of UPF		% of TEI		% of UPF	
	Mean	95 % CI*	Mean	95 % CI*	Mean	95 % CI*	Mean	95 % CI*
Unprocessed or minimally processed foods (UMF)	26	25·0, 26·1	–		29	28·7, 29·9	–	
Processed culinary ingredients (PCI)	3	3·2, 3·6	–		4	3·9, 4·3	–	
Processed foods (PF)	18	17·6, 18·5	–		16	16·1, 16·8	–	
Ultra-processed foods (UPF)	53	52·4, 53·6	–		50	49·6, 50·8	–	
Bread and breakfast foods	10	9·6, 10·3	19	18·4, 20·0	10	9·3, 9·8	19	19·0, 19·9
Candies, chocolate, flour-based sweets	10	9·3, 9·9	18	17·1, 18·0	9	8·7, 9·3	17	16·9, 18·0
Processed animal protein	8	8·0, 8·5	16	15·4, 16·3	7	7·0, 7·4	15	14·1, 15·0
Snacks and other savory foods	7	6·6, 7·0	13	12·6, 13·5	7	6·6, 6·9	14	13·2, 13·8
Processed beverages	7	6·3, 7·4	12	11·0, 12·5	5	4·4, 5·1	8	7·7, 8·7
Sauces, condiments and others	6	5·7, 6·2	11	11·0, 11·9	6	6·0, 6·4	13	12·6, 13·4
Processed milk and yogurt	4	3·6, 4·0	7	6·9, 7·7	5	4·6, 5·0	10	9·4, 10·2
Fruit juice	2	1·8, 2·1	4	3·6, 4·1	2	1·8, 2·0	4	3·7, 4·2

TEI, total daily energy intake.

* Means (95 % CI) are presented.

Table 3 shows the mean nutrient intake in the participant’s diet, overall and across quantiles of the %UPF. The population mean energy intake was 1899 and 1622 kcal per day for males and females, respectively. The mean dietary caloric contribution of UPF was 35 % (1st quantile) and 69 % (5th quantile) and 32 % (1st quantile) and 68 % (5th quantile), for males and females, respectively. The major changes in mean intake between participants in quantile 5 relative to those in quantile 1 for males and females, respectively, were for added sugar (+199 % and +202 %), vitamin K (–52 % and −60 %), folic acid (+26 % and +43 %), vitamin A (–38 % and −24 %), vitamin C (–34 % and −37 %), Mg (–33 % and −35 %) and fibre (–31 % and −37 %). In adjusted models, all mean nutrient-specific intake differs across the quantiles, except for total fat and protein intake in females. As the %UPF intake increases, the intake of carbohydrates, SFA, sugar, Na and folic acid significantly increases, while the intake of fibre and all vitamins and minerals decrease (all *P* values < 0·05) (Table 3).

**Table 3.** Nutritional profile according to caloric contribution of the ultra-processed foods in the Health and Retirement Study Population.

	Males N (%) 2,581 (45·34)								Females N (%) 3,639 (54·66)							
	Overall	UPFs (% of TEI)					*P*-value for trend		Overall	UPFs (% of TEI)					*P*-value for trend	
	Mean (SE)^†^	Q 1 (min· 10, max· 43)	Q 2 (min· 43, max· 50)	Q 3 (min· 50, max· 56)	Q 4 (min· 56, max· 62)	Q 5 (min· 62, max· 96)	Crude	Adjusted^‡^	Mean (SE)^†^	Q 1 (min· 8, max· 40)	Q 2 (min· 40, max· 47)	Q 3 (min· 47, max· 53)	Q 4 (min· 53, max· 61)	Q 5 (min· 61, max· 90)	Crude	Adjusted^§^
Calories (kcal)	1899 (16·9)	1757 (27·0)	1857 (42·2)	1917 (38·3)	1936 (38·5)	2013 (39·6)	<0·0001***	<0·0001***	1622 (10·7)	1536 (27·6)	1569 (26·2)	1596 (25·2)	1662 (28·2)	1744 (25·0)	<0·0001***	<0·0001***
Carbohydrate (kcal)	969 (10·2)	833 (19·4)	934 (24·7)	960 (22·4)	991 (18·2)	1114 (27·0)	<0·0001***	<0·0001***	846 (7·0)	760 (16·2)	797 (14·7)	818 (12·8)	868 (16·0)	982 (16·5)	<0·0001***	<0·0001***
Protein (kcal)	288 (2·9)	284 (4·7)	296 (7·3)	304 (6·4)	291 (6·1)	266 (5·5)	0·0152*	0·0083**	258 (2·0)	257 (4·4)	263 (4·4)	266 (4·8)	265 (4·9)	241 (3·9)	0·0344*	0·2219
Fat (kcal)	601 (5·7)	556 (9·8)	593 (15·1)	615 (12·2)	625 (15·8)	615 (12·5)	0·0009***	0·0035**	521 (3·9)	516 (11·8)	508 (8·8)	520 (9·7)	532 (9·8)	526 (9·0)	0·1992	0·0550
Saturated fatty acids (kcal)	201 (1·9)	173 (3·3)	196 (5·1)	205 (4·2)	212 (5·2)	218 (4·5)	<0·0001***	<0·0001***	171 (1·4)	149 (3·2)	165 (3·2)	175 (3·6)	182 (3·3)	186 (3·1)	<0·0001***	<0·0001***
Monounsaturated fatty acids (kcal)	220 (2·3)	217 (5·0)	219 (6·2)	224 (4·7)	226 (6·3)	216 (4·5)	0·7614	0·7661	190 (1·6)	202 (5·3)	187 (3·4)	188 (3·7)	189 (3·6)	182 (3·4)	0·0095**	0·1526
Cholesterol (mg/1000 kcal)	125 (1·4)	137 (4·2)	130 (2·9)	129 (2·98)	125 (2·7)	107 (2·2)	<0·0001***	<0·0001***	124 (1·1)	136 (3·1)	132 (2·9)	128 (1·7)	123 (2·2)	101 (1·5)	<0·0001***	<0·0001***
Fibers (gm/1000 kcal)	11 (0·1)	12 (0·2)	12 (0·2)	11 (0·2)	10 (0·1)	8 (0·1)	<0·0001***	<0·0001***	12 (0·1)	15 (0·2)	13 (0·2)	12 (0·1)	11 (0·1)	9 (0·1)	<0·0001***	<0·0001***
Total sugars (mg/1000 kcal)	61 (0·5)	52 (1·0)	57 (1·0)	58 (0·8)	62 (0·9)	77 (1·3)	<0·0001***	<0·0001***	62 (0·4)	55 (0·7)	58 (0·6)	60 (0·7)	62 (0·8)	76 (1·4)	<0·0001***	<0·0001***
Added sugars (mg/1000 kcal)	35 (0·6)	19 (0·5)	27 (0·7)	32 (0·7)	39 (0·9)	58 (1·4)	<0·0001***	<0·0001***	32 (0·5)	18 (0·4)	25 (0·5)	29 (0·6)	35 (0·7)	54 (1·5)	<0·0001***	<0·0001***
Vitamin A (μg/1000 kcal)	454 (4·8)	556 (11·9)	491 (14·6)	458 (9·9)	436 (10·7)	341 (8·9)	<0·0001***	<0·0001***	549 (6·0)	691 (13·4)	606 (12·3)	565 (11·0)	490 (9·9)	398 (8·4)	<0·0001***	<0·0001***
Vitamin B1 (mg/1000 kcal)	0·78 (0·004)	0·81 (0·01)	0·83 (0·01)	0·81 (0·01)	0·77 (0·01)	0·69 (0·01)	<0·0001***	<0·0001***	0·82 (0·003)	0·86 (0·01)	0·85 (0·01)	0·84 (0·01)	0·82 (0·01)	0·73 (0·01)	<0·0001***	<0·0001***
Vitamin B2 (mg/1000 kcal)	1·08 (0·01)	1·23 (0·02)	1·16 (0·01)	1·12 (0·02)	1·02 (0·01)	0·91 (0·02)	<0·0001***	<0·0001***	1·13 (0·01)	1·24 (0·02)	1·20 (0·01)	1·17 (0·02)	1·09 (0·01)	0·96 (0·02)	<0·0001***	<0·0001***
Folic acid (μg/1000 kcal)	57 (0·7)	47 (1·2)	57 (1·4)	61 (1·3)	63 (1·3)	59 (1·3)	<0·0001***	<0·0001***	54 (0·5)	43 (1·4)	51 (1·1)	56 (0·9)	60 (0·9)	61 (1·1)	<0·0001***	<0·0001***
Vitamin B12 (μg/1000 kcal)	6·7 (0·1)	7·3 (0·3)	7·5 (0·3)	7·0 (0·3)	6·3 (0·3)	5·4 (0·2)	<0·0001***	<0·0001***	6·8 (0·1)	7·6 (0·3)	7·2 (0·3)	7·3 (0·3)	6·7 (0·3)	5·4 (0·2)	<0·0001***	<0·0001***
Vitamin C (mg/1000 kcal)	52 (0·8)	63 (2·0)	57 (1·9)	52 (1·3)	49 (1·3)	42 (1·4)	<0·0001***	<0·0001***	59 (0·7)	73 (2·1)	63 (1·3)	59 (1·3)	54 (1·2)	46 (1·1)	<0·0001***	<0·0001***
Vitamin D (IU/1000 kcal)	108 (1·1)	135 (4·2)	122 (3·9)	109 (3·0)	96 (2·5)	84 (2·2)	<0·0001***	<0·0001***	116 (1·4)	135 (3·8)	130 (3·0)	125 (3·2)	104 (2·1)	85 (2·3)	<0·0001***	<0·0001***
Vitamin K (μg/1000 kcal)	70 (1·3)	97 (4·0)	73 (2·5)	70 (1·8)	65 (2·3)	46 (1·3)	<0·0001***	<0·0001***	93 (1·7)	142 (4·9)	103 (3·2)	91 (2·7)	75 (2·1)	57 (1·8)	<0·0001***	<0·0001***
Calcium (mg/1000 kcal)	431 (2·9)	473 (9·9)	464 (8·8)	441 (6·6)	414 (6·8)	371 (7·0)	<0·0001***	<0·0001***	459 (3·6)	486 (8·2)	486 (7·1)	484 (8·7)	443 (6·7)	399 (6·2)	<0·0001***	<0·0001***
Iron (mg/1000 kcal)	8·9 (0·1)	9·3 (0·2)	9·5 (0·2)	9·2 (0·2)	8·6 (0·2)	7·9 (0·2)	<0·0001***	<0·0001***	9·3 (0·1)	10·2 (0·2)	9·5 (0·2)	9·5 (0·2)	9·1 (0·2)	8·0 (0·1)	<0·0001***	<0·0001***
Magnesium (mg/1000 kcal)	168 (1·1)	202 (2·8)	182 (1·6)	169 (1·5)	157 (1·4)	136 (1·5)	<0·0001***	<0·0001***	184 (0·9)	224 (2·2)	196 (1·3)	184 (1·4)	169 (1·8)	146 (1·6)	<0·0001***	<0·0001***
Phosphorus (mg/1000 kcal)	665 (2·6)	742 (7·6)	710 (5·8)	682 (5·6)	640 (4·8)	563 (5·1)	<0·0001***	<0·0001***	702 (3·1)	772 (6·1)	743 (5·3)	725 (6·5)	680 (6·2)	596 (6·1)	<0·0001***	<0·0001***
Potassium (mg/1000 kcal)	1555 (7·8)	1799 (18·9)	1674 (13·1)	1586 (13·0)	1463 (12·2)	1280 (11·4)	<0·0001***	<0·0001***	1706 (7·6)	1979 (18·5)	1822 (12·5)	1743 (12·0)	1602 (12·1)	1392 (13·9)	<0·0001***	<0·0001***
Sodium (mg/1000 kcal)	1069 (5·1)	977 (12·9)	1071 (11·0)	1115 (9·8)	1123 (9·9)	1056 (13·1)	<0·0001***	<0·0001***	1055 (5·1)	945 (10·9)	1050 (8·6)	1094 (8·7)	1128 (10·6)	1061 (10·2)	<0·0001***	<0·0001***
Zinc (mg/1000 kcal)	5·47 (0·03)	5·71 (0·06)	5·66 (0·06)	5·67 (0·05)	5·46 (0·05)	4·88 (0·06)	<0·0001***	<0·0001***	5·69 (0·02)	5·93 (0·05)	5·89 (0·05)	5·86 (0·06)	5·72 (0·05)	5·09 (0·05)	<0·0001***	<0·0001***
Copper (μg/1000 kcal)	0·75 (0·01)	0·80 (0·02)	0·80 (0·02)	0·76 (0·01)	0·74 (0·01)	0·64 (0·01)	<0·0001***	<0·0001***	0·80 (0·01)	0·90 (0·01)	0·83 (0·01)	0·81 (0·01)	0·77 (0·01)	0·70 (0·01)	<0·0001***	<0·0001***

Abbreviations: UPFs, ultra-processed foods; TEI, total daily energy intake; Q, quantile; kcal, kilocalories; mg, milligram; gm, gram; μg, microgram; IU international unit.

† Means (standard errors) are presented.

‡ linear regression models were adjusted for age, race, years of education, total net income, food security, physical activity, smoking, vegetarian diet, cardiovascular diseases, and diabetes.

§ linear regression models were adjusted for age, race, years of education, total net income, food security, receive foods aids, body mass index, physical activity, smoking, vegetarian diet, cardiovascular diseases, and hypertension.

*P<0·05, **P<0·01, ***P<0·001.

Linear regression models were conducted to explore the socio-demographic and lifestyle factors associated with %UPF (Table 4). Among males, race was a predictive factor of %UPF, where Hispanic Americans reported lower intake compared with their White counterparts (*β* = −5·96 (95 % CI −8·10, −3·81)). Also, reporting adherence to a vegetarian diet (*β* = −6·46 (95 % CI −10·55, −2·37)), Mediterranean diet (*β* = −5·19 (95 % CI −6·34, −4·04)) and being physically active (*β* = −2·57 (95 % CI −4·47, −0·67)) were associated with lower %UPF intake. Income was inversely associated with %UPF (*β* = −0·004 (95 % CI −0·005, −0·003)) and having very low food security was associated with higher %UPF intake (*β* = 4·38 (95 % CI 1·01, 7·74)). Receiving a diagnosis of CVD (*β* = 2·03 (95 % CI 0·89, 3·17)) or diabetes (*β* = −1·53 (95 % CI −2·84, −0·23)) was associated with %UPF. Among females, similar associations were seen in the fully adjusted models with a slight change in the beta estimate. Moreover, older age was associated with higher %UPF intake (*β* = 0·09 (95 % CI 0·04, 0·14)) and every non-white race reported a lower intake of %UPF compared with their White counterparts (specifically, Hispanic (*β* = −6·68 (95 % −8·40, −4·96)), others (*β* = −4·87 (95 % −7·90, −1·84)) and African Americans (*β* = −3·29 (95 % CI −4·88, −1·70))). Active smoking (*β* = 3·86 (95 % CI 2·08, 5·64)) was correlated with %UPF among females.

**Table 4.** Sociodemographic and psychosocial determinants of the ultra-processed foods consumption of Health and Retirement Study Population

	Males N (%) 2,581(45·34)				Females N (%) 3,639 (54·66)			
	Bivariate		Multivariable^†^		Bivariate		Multivariable^†^	
	β (95 % CI)^‡^	*P*-value	β (95 % CI)^‡^	*P*-value	β (95 % CI)^‡^	*P*-value	β (95 % CI)^‡^	*P*-value
Age (years)	−0·04 (−0·10, 0·01)	0·0916	−0·055 (−0·112, −0·001)	0·0552	0·058 (0·003, 0·114)	0·0401*	0·058 (0·004, 0·113)	0·0372*
Race, % (Ref·= White)		<0·0001***		<0·0001***		<0·0001***		<0·0001***
African American	−0·35 (−2·42, 1·73)	0·7388	−1·48 (−3·71, 0·75)	0·1891	−1·52 (−3·12, 0·09)	0·0632	−3·24 (−4·91, −1·57)	0·0003***
Hispanic	−5·10 (−7·21, −2·98)	<0·0001***	−6·27 (−8·42, −4·11)	<0·0001***	−6·25 (−7·98, −4·52)	<0·0001***	−7·54 (−9·21, −5·86)	<0·0001***
Other	−4·71 (−9·61, 0·20)	0·0596	−4·46 (−9·04, 0·12)	0·0561	−5·73 (−8·68, −2·77)	0·0003***	−5·26 (−8·16, −2·35)	0·0006***
Marital status, % (Ref·= Married)		0·0557		0·3911		0·2125		0·5735
Separated/divorced/widowed	1·11 (−0·63, 2·85)	0·2067	0·13 (−1·38, 1·63)	0·8642	0·95 (−0·19, 2·10)	0·1011	−0·58 (−1·74, 0·58)	0·3186
Never married	2·84 (0·47, 5·20)	0·0198*	1·52 (−0·72, 3·75)	0·1800	0·11 (−2·32, 2·54)	0·9273	−0·01 (−2·25, 2·23)	0·9910
Education level, % (Ref·= Less than high school)		0·0007***		0·1474		<0·0001***		0·0004***
High school grad	1·32 (−0·37, 3·01)	0·1237	−0·06 (−1·59, 1·46)	0·9341	1·57 (−0·29, 3·43)	0·0967	0·78 (−0·98, 2·53)	0·3789
Some college/ grad	−0·49 (−2·14, 1·16)	0·5528	−0·95 (−2·64, 0·73)	0·2617	−1·04 (−3·30, 1·22)	0·3615	−0·67 (−2·89, 1·55)	0·5506
Post-college	−2·35 (−4·17, −0·53)	0·0123*	−1·929 (−3·861, −0·003)	0·0504	−3·82 (−6·27, −1·37)	0·0028**	−2·50 (−4·81, −0·20)	0·0337*
Total net wealth (US $)	−0·005 (−0·007, −0·004)	<0·0001***	−0·005 (−0·006, −0·003)	<0·0001***	−0·006 (−0·008, −0·004)	<0·0001***	−0·005 (−0·007, −0·003)	<0·0001***
Food insecurity, % (Ref·= Food security)		0·0060**		0·0458*		0·0081**		0·0050**
Low food security	1·00 (−1·43, 3·44)	0·4131	0·89 (−1·38, 3·17)	0·4348	0·77 (−1·73, 3·27)	0·5391	0·29 (−2·05, 2·63)	0·8068
Very low food security	4·56 (1·80, 7·31)	0·0016**	4·38 (0·94, 7·81)	0·0134*	4·09 (1·55, 6·63)	0·0021**	4·52 (1·83, 7·22)	0·0014**
Receiving free or subsidized foods, %	1·11 (−0·66, 2·87)	0·2141	−0·72 (−2·58, 1·14)	0·4409	1·88 (0·15, 3·61)	0·0333*	0·16 (−1·86, 2·19)	0·8734
Physical activity, % (Ref= Hardly ever or never)		<0·0001***		0·0006***		<0·0001***		<0·0001***
A few times/ month	−0·95 (−3·79, 1·90)	0·5074	−0·34 (−3·05, 2·38)	0·8049	−0·35 (−2·19, 1·48)	0·7021	0·16 (−1·70, 2·02)	0·8640
Once a week or more	−4·53 (−6·49, −2·57)	<0·0001***	−3·16 (−5·18, −1·14)	0·0027**	−4·75 (−6·24, −3·27)	<0·0001***	−3·31 (−4·70, −1·92)	<0·0001***
Smoking status, %	3·08 (1·27, 4·90)	0·0013**	1·11 (−0·79, 3·01)	0·2449	5·55 (3·80, 7·30)	<0·0001***	4·81 (3·01, 6·62)	<0·0001***
BMI, % (Ref·= Normal weight)		0·2306		0·3817		0·0006***		0·0009***
Underweight	−9·96 (−24·31, 4·39)	0·1701	−10·92 (−26·22, 4·38)	0·1585	3·91 (−0·86, 8·67)	0·1060	−1·90 (2·75, −6·61)	0·4163
Overweight	−0·45 (−2·17, 1·27)	0·6054	−0·12 (−1·76 1·51)	0·8801	−0·40 (−1·82, 1·02)	0·5756	−0·67 (−1·92, 0·59)	0·2913
Obese	0·69 (−1·04, 2·42)	0·4272	0·61 (−1·31, 2·53)	0·5290	2·07 (0·61, 3·54)	0·0062**	1·70 (0·31, 3·08)	0·0175*
Vegetarian diet, %	−8·32 (−12·99, −3·64)	0·0008***	−7·24 (−11·53, −2·95)	0·0013*	−8·18 (−11·78, −4·59)	<0·0001***	−6·42 (−9·72, −3·12)	0·0003***
Cardiovascular disease, %	2·22 (1·10, 3·35)	0·0002***	2·11 (0·93, 3·28)	0·0007***	1·39 (0·02, 2·76)	0·0465*	−0·36 (−1·76, 1·04)	0·6118
Hypertension, %	0·17 (−0·97, 1·31)	0·7699	−0·18 (−1·38, 1·02)	0·7621	1·16 (0·12, 2·20)	0·0298*	−0·01 (−1·00, 0·98)	0·9826
Diabetes, %	−1·40 (−2·77, −0·03)	0·0454*	−2·01 (−3·37, −0·65)	0·0044**	−1·13 (−2·45, 0·19)	0·0908	−2·27 (−3·43, −1·11)	0·0002***

Abbreviations: UPFs, ultra-processed foods; TEI, total daily energy intake; UPFs, ultra-processed foods; Ref·, reference; BMI, Body Mass Index.

† All covariates are included in the multivariable models.

‡ Beta estimates (β) (95% confidence intervals (CIs)) are presented.

*P<0·05, **P<0·01, ***P<0·001.

## Discussion

In the current study, %UPF consumption and its cross-sectional associations with nutrient intake and socio-demographic and lifestyle characteristics were evaluated among a representative sample of USA middle-older adults. Approximately 50 % of the total energy intake comes from UPF, followed by unprocessed and minimally processed foods. A higher intake of %UPF was associated with lower diet quality. Lastly, the data revealed that %UPF intake was linked to a few socio-demographic and lifestyle characteristics among the study population, and some of these associations were sex-specific.

UPF account for half or more of the caloric share among USA middle-older adults, consistent with studies conducted in the USA^(42–44)^, UK^(28,45)^ and Canada^(46)^. However, the current study’s estimate is higher than %UPF reported in other studies in Canada^(26,27)^, Australia^(47)^, Portugal^(48)^, Brazil^(49,50)^ and France^(25)^. It is worth noting that country differences in %UPF intake have been noticed before^(51)^. For example, Martini et al. conducted a meta-analysis of nationally representative samples from fourteen countries and showed that the USA was among the countries with the highest %UPF intake^(51)^. Indeed, food culture was proposed as a factor justifying the country differences in %UPF. Food culture promoting home cooking and handmade dishes could be a protective factor against the UPF via encouraging subjects to consume more of minimally processed foods^(25)^. Furthermore, sample characteristics could explain lower %UPF intake in some studies. To illustrate, minimally processed foods and UPF accounted for 64·7 % and 17·7 %, respectively, among Brazilian farmers in the rural area^(49)^, which was acknowledged to be lower even than the national %UPF estimate (20 %)^(49)^.

In the current study, the two most frequent UPF subcategories were (1) bread and breakfast foods and (2) candies, chocolate and flour-based sweets; they collectively account for more than third of the UPF intake. Consistent with this observation, bread^(44,46,50)^ and other flour-based products^(48,49,51)^ were reported to be the highest UPF subcategories in other studies. Other studies, however, reported different top contributors than flour-based products^(25,26,48)^, which could be influenced by cultural differences. For example, fruit and vegetables sourced from ready-to-eat products in France^(25)^, dairy products in Australia^(48)^ and soft drinks and fruit juices and drinks in Brazil^(26)^ were the top UPF drivers. Not only are there cultural differences in the UPF sub-categories, but sex differences have also been observed in other studies. Despite higher intake among Brazilian women from all UPF sub-categories, soft drinks were consumed more by their male counterparts^(50)^. Besides, evidence from twenty-two European countries showed a higher fine bakery consumption in women, but a higher sausage consumption in men^(52)^. Among Norwegian men, higher intakes from ultra-processed dinner products and fast foods were reported compared with women^(53)^. Further studies are needed to explore the sex-specific differences in the UPF sub-categories to shed light on the effective strategies for reducing the UPF consumption.

We detected a linear relationship between %UPF and nutrient intake in this study, which is consistent with previous research. Positive trends for calories^(25,49)^, carbohydrates^(25,26,42)^, saturated fat^(26,42,51)^, sugar^(25,26,42)^ and inverse trends for fibre^(25,42,49,51)^, vitamin A^(26,42,49,51)^, vitamin B_12_^(26,51)^, vitamin C^(25,26,42,49,51)^, Ca^(25,26,42,49)^, iron^(26,49)^, Mg^(26,51)^, phosphorous^(26,42,49)^ and potassium^(26,42,49,51)^ were documented. In fact, the decline in diets’ nutritious value has been one of the plausible proposed mechanisms connecting UPF with negative health outcomes. Because maintaining good nutrition is an integral component of healthy ageing^(6)^, the current study strengthens the call for interventions aiming to reduce the UPF consumption among middle-older adults in order to improve diet quality.

%UPF and age, as well as food insecurity, were found to have positive associations. The weak significant positive association for age among females contradicts other studies^(25–28,43,47,48,50)^. However, because these studies included subjects aged 1·5 to ≥ 80 years old, their findings may not capture the subtle increase in UPF among the older adult’s population. On the other hand, it is worth noting that UPF intake was reported to be higher among the older age strata compared with the younger adult strata^(26,47)^. For example, UPF intake was 42·6 % in Canadians aged ≥ 65 years *v.* 42·6 % in those aged 51–64 years^(26)^ and 38·4 % in Australians aged ≥ 71 years *v.* 35·5 % in those aged 51–70 years^(47)^. Multiple factors could influence older adults’ foods choices and dietary habits, which could explain the increase in UPF intake among older adults. For example, food affordability, including the cost of foods, eating out and transportation activities for food^(54)^, has been reported as a concern among USA older adults^(54)^. Food affordability is a crucial factor in food security. Food insecurity is a pressing public health challenge for USA older adults^(10,11)^. Previous research has shown that food insecurity is not only associated with poor diet quality^(11)^ but also with higher UPF intake^(55)^. Given that UPF are energy dense at a lower cost^(56)^, they were more likely to be consumed among food insecure older adults. Future interventions aiming to mitigate the UPF consumption among older adults should plan to address food insecurity to yield effective outcomes.

The results showed that income, physical activity and following a vegetarian diet or Mediterranean diet were inversely associated with %UPF in males and females. Following an overall healthy lifestyle pattern was associated with higher diet quality, and it has been shown that physical inactivity^(27)^ and low income^(25,43,47)^ were associated with higher %UPF intake. We acknowledge the potential of reverse causation in our inverse cross-sectional association between %UPF and self-reported diabetes diagnosis as it might be partially explained by receiving dietary education for blood glucose management, where the emphasis on whole and minimally processed foods intake is a hallmark. The current study showed that White Americans had higher %UPF intake compared with other races/ethnicities; this observation is consistent with other studies that demonstrated higher intake of highly processed foods^(43)^ of highly processed foods among White American adults than other racial/ethnic groups.

Among the study sample, female smokers were more likely to report higher %UPF consumption, which is consistent with other studies^(25,27,28)^. Females with obesity tend to consume more %UPF than their normal weight counterparts. Previous studies showed that UPF were positively associated with overweight^(25)^ and obesity^(25,27,28)^. Longitudinal studies are warranted to examine the association between UPF and body weight among older adults. Among males only, UPF was positively associated with CVD diagnoses. Further studies are needed to examine the sex-specific determinants of UPF among older adults and prospective designs would yield robust evidence.

Using a representative sample of USA middle-older adults was a major strength for contributing to the current knowledge. Another strength was the use of the Nova system framework to classify the diet quality. Not only has the Nova system been a commonly used food processing classification in the literature^(12)^ and an applicable tool in epidemiological public health and nutrition studies^(47)^ but also a measure to guide food-related polices due to its focus on food’s processing and industry^(48)^. Moreover, a sex-stratified analysis was conducted because of the documented evidence for the sex difference in UPF consumption^(25–28)^. The %UPF intake was assessed as a categorical variable through quantiles-based analysis and as a continuous variable; however, we support the call for standardisation in estimating the association for UPF intake^(57)^ to enhance the comparability between studies.

However, the study has some limitations that are worth discussing. Causality cannot be interfered due to the cross-sectional design. It is noteworthy that the dietary intake assessed in 2013 was the only data available thus far among this population, which might not precisely reflect the current intake. Nevertheless, the current analysis could be used as a reference to track the change in %UPF consumption, and as a source for guiding future studies focusing on the identified determinants for mitigating UPF consumption among USA middle-older adults. Furthermore, a couple of limitations inherited due to the assessment of UPF should be acknowledged. We confirmed that FFQ used has been validated to capture self-reported calories and nutrients; however, thus far this FFQ has not been validated yet to assess UPF^(37)^. Another limitation pertained to the FFQ, there is potential concern for misclassifying UPF^(14–16)^ because of lack of information in the FFQ on cooking methods, brands and other information that might help in accurately classifying the food items based on the degree of food process^(37)^. In addition, classifying each item entirely into a single Nova group ignores the fact that composite dishes are made with different proportions of multiple Nova groups. Having said that, standard dietary assessment tools – FFQ, food records or 24-h recalls – have been used before to assess UPF^(37)^. As an attempt to reduce these potential misclassifications mentioned previously, four methods of classifying UPF that were used in the current study, where applied by other researchers^(36,37)^ (online Supplementary Table 1). However, future studies are warranted to explore ways to reduce the measurement errors in estimating UPF from population-based dietary assessment tools. Also, further revisions to the Nova classification system are needed to address the limitations identified earlier in classifying foods^(16)^.

In conclusion, the study revealed higher %UPF intake among a nationally representative sample of USA middle-older adults, as well as the most common UPF groups consumed. Furthermore, because %UPF intake was associated with lower nutrient quality among older adults, efforts are needed for reducing UPF intake to promote healthy ageing. The predictors associated with %UPF were identified, and food insecurity, an ongoing public health challenge for older adults, was one of the determinants. Because of the ubiquity of UPF, and the documented link between UPF and impaired health outcomes for older adults^(17–24)^, our findings have public health relevance for food policymakers to intervene with efforts to improve the accessibility and consumption of fresh and minimally processed foods while aiming to mitigate the food insecurity. Furthermore, the findings highlighted the inequalities across middle-older adults in regard to the UPF.

## Supporting information

Aljahdali et al. supplementary material Aljahdali et al. supplementary material

## Supplementary material

For supplementary material/s referred to in this article, please visit https://doi.org/10.1017/S0007114523003033
