# Supplementary material for: Ultra-processed foods consumption among a USA representative sample of middle-older adults: a cross-sectional analysis
Source: Br J Nutr. 2024 Jan 4;131(8):1461–72. doi: 10.1017/S0007114523003033 (PMC10950447; doi:10.1017/S0007114523003033)
Supplement: Aljahdali et al. supplementary material [file S0007114523003033sup001.docx]

# Supplementary Table 1: Nova group classification for the food items listed in the food frequency questionnaires used in the Health and Retirement Study:

| **Food item** | **Nova** | **Sensitivity analyses** | | | |
| --- | --- | --- | --- | --- | --- |
|  |  | **1** | **2** | **3** | **4** |
| Skim milk | 1 |  |  |  |  |
| 1% or 2% milk | 1 |  |  |  |  |
| Whole milk | 1 |  |  |  |  |
| Soy milk | 4 | 1 |  | 1 |  |
| Cream, e.g., in coffee, whipped or sour cream | 2 |  | 4 |  |  |
| Non-dairy coffee whitener | 2 | 4 | 4 | 4 |  |
| Frozen yogurt, sherbert or low-fat ice cream | 4 |  |  |  |  |
| Regular ice cream | 4 |  |  |  |  |
| Flavored yogurt, sweetened with fruit or other flavoring | 4 |  |  |  |  |
| Yogurt, low carb, artificially sweetened or plain | 4 | 1 | 1 |  |  |
| Cottage or ricotta cheese | 3 |  |  |  |  |
| Cream cheese | 3 | 4 | 4 | 4 |  |
| Other cheese, e.g., American, cheddar, etc., plain or as part of a dish | 3 |  |  |  |  |
| Pure butter, added to food or bread; exclude use in cooking | 2 |  |  |  |  |
| “Spreadable butter” –butter/oil blend, added to food or bread; exclude use in cooking | 2 | 4 | 4 | 4 |  |
| Margarine or spread, added to food or bread; exclude use in cooking | 4 |  |  |  |  |
| Raisins or grapes | 1 |  |  |  |  |
| Prunes or dried plums | 3 | 1 | 1 | 1 |  |
| Prune juice | 4 | 1 | 1 | 1 | 3 |
| Bananas | 1 |  |  |  |  |
| Melon (cantaloupe, honeydew, watermelon) | 1 |  |  |  |  |
| Avocado | 1 |  |  |  |  |
| Applesauce | 3 | 1 | 4 | 1 |  |
| Fresh apples or pears | 1 |  |  |  |  |
| Apple juice or cider | 4 | 1 | 1 | 3 |  |
| Oranges | 1 |  |  |  |  |
| Orange juice - calcium or vitamin D fortified | 4 | 1 | 1 | 1 |  |
| Orange juice - regular (not fortified) | 1 |  |  |  |  |
| Grapefruit | 1 |  |  |  |  |
| Grapefruit juice | 1 |  |  |  |  |
| Other fruit juices (grape, cranberry) | 4 | 1 | 1 | 1 |  |
| Strawberries, fresh, frozen, or canned | 1 |  |  |  |  |
| Other berries (e.g., blueberries, raspberries, blackberries) fresh, frozen, or canned | 1 |  |  |  |  |
| Peaches or plums (fresh or canned) | 1 |  |  | 4 | 3 |
| Apricots (fresh, canned, or dried) | 1 |  |  |  |  |
| Tomatoes | 1 |  |  |  |  |
| Onions as a garnish or in salad | 1 |  |  |  |  |
| Onions as cooked vegetable, rings, or soup | 1 |  |  |  |  |
| Tomato or V8 juice | 4 | 1 | 1 | 3 |  |
| Tomato sauce e.g., spaghetti sauce | 4 | 3 |  | 3 |  |
| Salsa, picante or taco sauce | 4 | 1 | 1 |  |  |
| Tofu, soy burgers, soybeans, miso, or other soy protein | 1 | 3 | 3 | 4 |  |
| Green beans or string beans | 1 |  |  |  |  |
| Broccoli | 1 |  |  |  |  |
| Cabbage or cole slaw | 1 |  |  |  |  |
| Cauliflower | 1 |  |  |  |  |
| Brussels sprouts | 1 |  |  |  |  |
| Carrots, raw | 1 |  |  |  |  |
| Carrots, cooked or carrot juice | 1 |  |  |  |  |
| Corn (ear, frozen, or canned) | 1 |  |  |  |  |
| Peas or lima beans (fresh, frozen, or canned) | 1 |  |  |  |  |
| Mixed or stir-fry vegetables, or vegetable soup | 1 |  | 4 | 3 | 3 |
| Beans or lentils, baked dried or soup | 1 |  |  |  |  |
| Dark orange (winter) squash | 1 |  |  |  |  |
| Eggplant, zucchini, or other summer squash | 1 |  |  |  |  |
| Yams or sweet potatoes | 1 |  |  |  |  |
| Spinach, cooked | 1 |  |  |  | 3 |
| Spinach, raw as in salad | 1 |  |  |  |  |
| Kale, mustard, or chard greens | 1 |  |  |  |  |
| Iceberg or head lettuce | 1 |  |  |  |  |
| Romaine or leaf lettuce | 1 |  |  |  |  |
| Celery | 1 |  |  |  |  |
| Peppers: green, yellow, or red | 1 |  |  |  |  |
| Egg Beaters or egg whites only | 1 |  |  |  |  |
| Omega-3 fortified eggs, including yolk | 1 |  |  |  |  |
| Regular eggs, with yolk | 1 |  |  |  |  |
| Bacon | 3 | 4 | 4 | 4 |  |
| Chicken or turkey sandwich or frozen dinner | 4 | 1 |  | 1 |  |
| Other chicken or turkey, with skin - including ground | 3 | 1 | 1 | 1 |  |
| Other chicken or turkey, including ground without skin | 3 | 1 | 1 | 1 |  |
| Beef or pork hot dogs | 4 |  |  |  |  |
| Chicken or turkey hot dogs or sausage | 4 |  |  |  |  |
| Salami, bologna, or other processed meat sandwiches | 4 |  |  |  |  |
| Other processed meats e.g., sausage, kielbasa, etc. | 4 |  |  |  |  |
| Hamburger, lean or extra lean | 4 | 1 | 1 | 1 | 1 |
| Hamburger, regular | 4 | 1 | 1 |  |  |
| Beef, pork, or lamb as a sandwich or mixed dish, e.g., stew, casserole, lasagna, frozen dinner, etc. | 4 | 1 |  | 1 |  |
| Pork as a main dish, e.g., ham or chops | 4 | 1 | 1 | 1 |  |
| Beef or lamb as a main dish, e.g., steak, roast | 3 | 1 | 1 | 1 |  |
| Liver: beef, calf or pork | 3 | 1 | 1 | 1 |  |
| Liver: chicken or turkey | 3 | 1 | 1 | 1 |  |
| Canned tuna fish | 3 |  |  |  |  |
| Breaded fish cakes, pieces, or fish sticks (store bought) | 4 |  |  |  |  |
| Shrimp, lobster, scallops, clams as a main dish | 3 | 1 | 1 | 1 |  |
| Dark meat fish, e.g., tuna steak, mackerel, salmon, sardines, bluefish, swordfish | 3 | 1 | 1 | 1 |  |
| Other fish, e.g., cod, haddock, halibut | 3 | 1 | 1 | 1 |  |
| Cold breakfast cereal | 4 |  |  |  |  |
| Cooked oatmeal/cooked oat bran | 1 |  |  |  |  |
| Other cooked breakfast cereal | 4 | 1 | 1 | 1 | 1 |
| White bread, including pita bread | 4 |  |  |  |  |
| Rye or Pumpernickel bread | 4 |  |  |  |  |
| Whole wheat, oatmeal, other whole grain bread | 4 |  |  |  |  |
| Bagels, English muffins, or rolls | 4 |  |  |  |  |
| Muffins or biscuits | 3 | 4 | 4 | 4 | 4 |
| Brown rice | 1 |  |  |  | 3 |
| White rice | 1 |  |  |  | 3 |
| Pasta, e.g., spaghetti, noodles, couscous, etc. | 4 | 1 | 1 | 1 |  |
| Tortillas | 4 | 3 | 3 | 3 | 3 |
| Other grains, e.g., bulgar, kasha, buckwheat, etc. | 1 |  |  |  |  |
| Pancakes or waffles | 3 | 1 | 4 | 1 |  |
| French fried potatoes | 3 | 4 | 4 | 4 |  |
| Potatoes, baked, boiled, or mashed | 1 |  |  |  |  |
| Potato chips or corn/tortilla chips | 4 | 3 |  | 3 |  |
| Crackers, regular or low fat | 4 |  |  |  |  |
| Crackers, whole wheat or whole grain, e.g., Triscuits | 4 |  |  |  |  |
| Other crackers | 4 |  |  |  |  |
| Pizza | 4 |  |  |  |  |
| Low-calorie beverage with caffeine, e.g., Diet Coke, Diet Mt. Dew | 4 |  |  |  |  |
| Other low-calorie beverages without caffeine, e.g., Diet 7-up | 4 |  |  |  |  |
| Carbonated beverage with caffeine and sugar, e.g., Coke, Pepsi, Mt. Dew, Dr. Pepper | 4 |  |  |  |  |
| Other carbonated beverages with sugar, e.g., 7-up, Root Beer, Ginger Ale | 4 |  |  |  |  |
| Other sugared beverages: Punch, lemonade, sports drinks, or sugared iced tea | 4 |  |  |  |  |
| Beer regular | 3 |  |  |  |  |
| Light beer, e.g., Bud Light | 3 |  |  |  |  |
| Red wine | 3 |  |  |  |  |
| White wine | 3 |  |  |  |  |
| Liquor, e.g., whiskey, gin, etc. | 4 |  |  |  |  |
| Herbal tea or decaffeinated tea | 1 |  |  |  |  |
| Tea with caffeine, including green tea | 1 |  |  |  |  |
| Decaffeinated coffee | 1 |  |  |  |  |
| Coffee with caffeine | 1 |  |  |  |  |
| Dairy coffee drink (hot/cold) e.g., cappuccino | 1 | 4 | 4 | 4 | 4 |
| Milk chocolate (bar or packet), (e.g., Hershey’s, M&M’s) | 4 |  |  |  |  |
| Low carb bars, e.g., Atkins, Zone, South Beach | 4 |  |  |  |  |
| Oat bran, added to food | 1 |  |  |  |  |
| Other bran (wheat, etc.), added to food | 1 |  |  |  |  |
| Wheat germ | 1 |  |  |  |  |
| Chowder or cream soup | 4 | 1 | 1 |  |  |
| Ketchup or red chili sauce | 4 |  |  |  |  |
| Salt added at table | 2 |  |  |  |  |
| Sugar added to beverages or food | 2 |  |  |  |  |
| Splenda | 4 |  |  |  |  |
| Other artificial sweetener | 4 |  |  |  |  |
| Garlic | 1 |  |  |  |  |
| Low fat or fat free mayonnaise | 4 |  |  |  |  |
| Regular mayonnaise | 4 |  |  |  |  |
| Salad dressing | 4 | 1 | 1 |  |  |
| Olive oil added to food or bread; exclude use in cooking | 2 |  |  |  |  |
| Dark chocolate, (e.g., Hershey’s Dark or Dove Dark) | 4 |  |  |  |  |
| Candy bars, (e.g., Snickers, Milky Way, Reeses) | 4 |  |  |  |  |
| Candy without chocolate (e.g., pack mints, Lifesavers) | 4 |  |  |  |  |
| Jams, jellies, preserves, syrup, or honey | 4 |  |  |  |  |
| Peanut butter | 4 | 1 | 1 | 3 |  |
| Fat free or light popcorn | 4 |  |  |  |  |
| Regular popcorn | 4 | 3 |  | 3 |  |
| Pretzels | 4 | 3 | 3 | 3 | 3 |
| Cookies, fat free or reduced | 4 |  |  |  |  |
| Cookies, other ready-made | 4 |  |  |  |  |
| Cookies, home baked | 3 | 1 | 1 | 1 | 4 |
| Brownies | 4 |  |  |  |  |
| Doughnuts | 4 |  |  |  |  |
| Cake, home baked | 3 | 1 | 1 | 1 |  |
| Cake, ready-made | 4 |  |  |  |  |
| Pie homemade | 3 | 1 | 4 | 1 |  |
| Pie, ready-made | 4 |  |  |  |  |
| Sweet roll, coffee cake or other pastry, fat free or reduced fat | 4 |  |  |  |  |
| Sweet roll, coffee cake or other ready-made pastry | 4 |  |  |  |  |
| Sweet roll, coffee cake or other pastry, home baked | 4 | 1 | 1 | 1 | 3 |
| Peanuts | 1 |  |  |  |  |
| Walnuts | 1 |  |  |  |  |
| Other nuts | 1 |  |  |  |  |
| Breakfast bars, e.g., Nutrigrain, granola, Kashi | 4 |  |  |  |  |
| Energy bars, e.g., Clif, Luna, Glucerna, Powerbar | 4 |  |  |  |  |

Cells are shaded in gray have same Nova classification explained in Classification 1 column

1= Unprocessed or minimally processed foods (UMFs); 2= Processed culinary ingredients (PCIs); 3= Processed food (PFs); and 4= Ultra-processed food (UPFs)

# Supplementary Table 2: Grouping the foods frequencies questionnaire items:

| **Unprocessed or minimally processed foods (UMFs)** | | |
| --- | --- | --- |
| 1 | Milk and eggs | Milk (skim, 1% or 2%, whole), egg (eggbeaters or egg whites only, omega-3 fortified eggs, regular eggs) |
| 2 | Vegetables | Tomatoes, green beans/string beans, broccoli, cabbage or cole slaw, cauliflower, brussels sprouts, carrots, corn, peas or lima beans, mixed or stir-fry vegetables, vegetable soup  dark orange/winter squash, eggplant, zucchini, or other summer squash, yams or sweet potatoes, spinach, kale, mustard, or chard greens, iceberg or head lettuce, romaine or leaf lettuce, celery, peppers (green, yellow, or red), onions, garlic, Tofu, soy burgers, soybeans, miso, or other soy protein, beans or lentils |
| 3 | Fruit | Raisins or grapes, bananas, melon (cantaloupe, honeydew, watermelon), avocado, apples or pears, oranges and orange juice (regular), grapefruit and grapefruit juice, strawberries, other berries (blueberries, raspberries, blackberries), peaches or plums, apricots |
| 4 | Coffee and tea | Tea (herbal, decaffeinated, caffeinated), coffee (decaffeinated, caffeinated), dairy coffee drink (hot/cold) |
| 5 | Grains, cereals, nuts, and others | Oatmeal/cooked oat bran, other grains (e.g., bulgar, kasha, buckwheat, etc.), potatoes, oat bran, other bran, wheat germ, peanuts, walnuts, other nuts, rice (brown or white) |
| **Processed culinary ingredients (PCIs)** | | |
| 6 | Culinary ingredients | Cream (e.g., in coffee, whipped or sour cream), non-dairy coffee whitener, pure butter, spreadable butter –butter/oil blend, salt, sugar, olive oil |
| **Processed foods (PFs)** | | |
| 7 | Cheese and fermented beverages | Cheese (cottage or ricotta cheese, cream cheese, American, cheddar, etc.,), beer (light, regular), wine (red, white) |
| 8 | Animal protein | Bacon, chicken or turkey, with or without skin - including ground, beef or lamb as a main dish, liver (beef, calf, pork, chicken or turkey), canned tuna fish, shrimp, lobster, scallops, clams as a main dish, dark meat fish (e.g., tuna steak, mackerel, salmon, sardines, bluefish, swordfish), other fish (e.g., cod, haddock, halibut) |
| 9 | Sweetened fruit and homemade foods | Prunes or dried plums, applesauce, muffins or biscuits, pancakes or waffles, French fried potatoes, home baked deserts (cookies, cake, pie) |
| **Ultra-processed foods (UPFs)** | | |
| 10 | Processed animal protein | Chicken or turkey sandwich or frozen dinner, hot dogs (beef, pork, chicken or turkey), salami, bologna, or other processed meat sandwiches, other processed meats (e.g., sausage, kielbasa, etc.), hamburger (lean or extra lean, regular), beef, pork, or lamb as a sandwich or mixed dish, (e.g., stew, casserole, lasagna, frozen dinner, etc.), pork as a main dish, (e.g., ham or chops), breaded fish cakes, pieces, or fish sticks |
| 11 | Bread, and breakfast foods | Breakfast cereal (cold, cooked), bread (white – including pita bread, rye or pumpernickel, whole wheat, oatmeal, other whole grain), bagels, English muffins, or rolls, pasta (e.g., spaghetti, noodles, couscous, etc.), tortillas, breakfast bars, (e.g., Nutrigrain, granola, Kashi) |
| 12 | Processed beverages | Low-calorie beverage with and without caffeine, carbonated beverage with sugar and with or without caffeine, other sugared beverages (punch, lemonade, sports drinks, or sugared iced tea, liquor (e.g., whiskey, gin, etc.) |
| 13 | Sauces, condiment, and others | Margarine or spread, tomato or V8 juice, tomato sauce (e.g., spaghetti sauce), salsa, picante or taco sauce, ketchup or red chili sauce, Splenda, other artificial sweetener, mayonnaise (low fat or fat free, regular), salad dressing, jams, jellies, preserves, syrup, or honey, peanut butter |
| 14 | Processed milk and yogurt | Soy milk, frozen yogurt, sherbert or low-fat ice cream, regular ice, flavored yogurt, sweetened with fruit or other flavoring, yogurt, low carb, artificially sweetened or plain |
| 15 | Fruit juice | Juice (prune, apple juice or cider, organic- calcium or vitamin D fortified, others (grape, cranberry)) |
| 16 | Candies, chocolate, flour-based sweets | Low carb bars (e.g., Atkins, Zone, South Beach), energy bars (e.g., Clif, Luna, Glucerna, Powerbar), chocolate (milk and dark), candy bars, (e.g., Snickers, Milky Way, Reeses), candy without chocolate (e.g., mints, Lifesavers), cookies (fat free or reduced, other ready-made), brownies, doughnuts, Cake or pie, ready-made, sweet roll, coffee cake or other pastry (fat free or reduced fat, ready-made, home baked) |
| 17 | Snack and other savory foods | Potato chips or corn/tortilla chips, crackers (regular, or low fat, whole wheat or whole grain, others), pizza, chowder or cream soup, popcorn (fat free or light popcorn, regular), pretzels |

# Supplementary Table 3: Mean dietary caloric contribution Nova groups and subgroups among Health and Retirement Study Population:

|  | **Males N (%)**  2,581(45.34) | | **Females N (%)**  3,639 (54.66) | |
| --- | --- | --- | --- | --- |
|  | **% of TEI** | **% of Nova group** | **% of TEI** | **% of Nova group** |
|  | **Mean (95% CI)^1^** | **Mean (95% CI)^1^** | **Mean (95% CI)^1^** | **Mean (95% CI)^1^** |
| **Unprocessed or minimally processed foods (UMPs)** | 26 (25.0, 26.1) | - | 29 (28.7, 29.9) | **-** |
| Milk and eggs | 6 (5.8, 6.2) | 24 (23.0, 24.8) | 6 (5.5, 5.9) | 20 (19.0, 20.2) |
| Vegetables | 4 (3.9, 4.1) | 17(16.1, 17.0) | 5 (5.0, 5.4) | 19 (18.1, 19.0) |
| Fruit | 6 (5.9, 6.5) | 23 (22.5, 24.1) | 7 (7.2, 7.7) | 25 (24.6, 25.7) |
| Coffee and tea | 1 (0.7, 0.9) | 3 (2.7, 3.4) | 1 (1.1, 1.3) | 4 (3.6, 4.3) |
| Grains, cereals, nuts, and others | 9 (8.3, 8.8) | 33 (32.5, 33.9) | 10 (9.4, 10.0) | 33 (32.1, 33.3) |
| **Processed culinary ingredients (PCIs)** | 3 (3.2, 3.6) | - | 4 (3.94, 4.27) | **-** |
| Culinary ingredients | 3 (3.2, 3.6) | 100.00 |  |  |
| **Processed foods (PFs)** | 18 (17.6, 18.5) | - | 16 (16.1, 16.8) | **-** |
| Cheese and fermented beverages | 6 (5.4, 6.2) | 28 (26.3, 28.7) | 4 (3.8, 4.3) | 22 (21.3, 23.1) |
| Animal protein | 6 (5.8, 6.1) | 36 (34.8, 36.6) | 7 (6.5, 6.7) | 41 (40.4, 41.6) |
| Sweetened fruit and homemade food | 6 (6.1, 6.5) | 37 (35.7, 37.9) | 6 (5.7, 6.0) | 37 (36.0, 37.7) |

Abbreviations: TEI, total daily energy intake.

^1^ Means (95% confidence intervals (CIs)) are presented.
